# Altering the ribosomal subunit ratio in yeast maximizes recombinant protein yield

**DOI:** 10.1186/1475-2859-8-10

**Published:** 2009-01-29

**Authors:** Nicklas Bonander, Richard AJ Darby, Ljuban Grgic, Nagamani Bora, Jikai Wen, Saverio Brogna, David R Poyner, Michael AA O'Neill, Roslyn M Bill

**Affiliations:** 1 School of Life and Health Sciences, Aston University, Aston Triangle, Birmingham B4 7ET, UK; 2 School of Biosciences, University of Birmingham, Edgbaston, Birmingham B15 2TT, UK; 3 Department of Pharmacy and Pharmacology, University of Bath, Claverton Down, Bath BA2 7AY, UK

## Abstract

**Background:**

The production of high yields of recombinant proteins is an enduring bottleneck in the post-genomic sciences that has yet to be addressed in a truly rational manner. Typically eukaryotic protein production experiments have relied on varying expression construct cassettes such as promoters and tags, or culture process parameters such as pH, temperature and aeration to enhance yields. These approaches require repeated rounds of trial-and-error optimization and cannot provide a mechanistic insight into the biology of recombinant protein production. We published an early transcriptome analysis that identified genes implicated in successful membrane protein production experiments in yeast. While there has been a subsequent explosion in such analyses in a range of production organisms, no one has yet exploited the genes identified. The aim of this study was to use the results of our previous comparative transcriptome analysis to engineer improved yeast strains and thereby gain an understanding of the mechanisms involved in high-yielding protein production hosts.

**Results:**

We show that tuning *BMS1 *transcript levels in a doxycycline-dependent manner resulted in optimized yields of functional membrane and soluble protein targets. Online flow microcalorimetry demonstrated that there had been a substantial metabolic change to cells cultured under high-yielding conditions, and in particular that high yielding cells were more metabolically efficient. Polysome profiling showed that the key molecular event contributing to this metabolically efficient, high-yielding phenotype is a perturbation of the ratio of 60S to 40S ribosomal subunits from approximately 1:1 to 2:1, and correspondingly of 25S:18S ratios from 2:1 to 3:1. This result is consistent with the role of the gene product of *BMS1 *in ribosome biogenesis.

**Conclusion:**

This work demonstrates the power of a rational approach to recombinant protein production by using the results of transcriptome analysis to engineer improved strains, thereby revealing the underlying biological events involved.

## Background

Advances in understanding cellular function rely on improving our knowledge of protein behaviour, protein-protein interactions, and the complex interplay of proteins with other biomolecules. Whilst structures have been solved for many individual proteins, the challenge now is to expand this specific knowledge more generically to physiologically-important, difficult-to-study eukaryotic proteins and to understand the interplay between them in complex systems. Understanding the structure and function of human proteins, and particularly membrane proteins, will not only disclose the underlying structural basis of human function but is vital in the development of new drugs in the fight against human disease [1].

As they are not naturally highly abundant, membrane proteins and many soluble eukaryotic proteins must be over-produced for the detailed studies that will reveal their biochemical, functional and structural characteristics. Therefore obtaining high yields of functional, recombinant protein remains a major bottleneck in contemporary bioscience [2]. We have shown that the root of the problem is the host organism [3], and the lack of knowledge about the intricate cellular biology within. Typically eukaryotic protein production experiments have relied on varying either promoter and fusion tag combinations in expression constructs [4] or culture process parameters such as pH, temperature and aeration [5] to enhance yields. These approaches require repeated rounds of trial-and-error optimization and cannot provide a mechanistic insight into the biology of recombinant protein production as only external parameters are varied. This is also true of approaches which rely on the mutation of the protein target to improve its production yields [6]. The genomics revolution, however, has allowed us to take a broader but still rational approach to such optimization, which we previously adopted for recombinant membrane protein production [3] where we reported 39 host cell (*S. cerevisiae*) genes whose expression was significantly altered when the glycerol facilitator, Fps1, was produced under high-yielding conditions (20°C, pH5) compared to low-yielding standard growth conditions (30°C, pH5). Although similar studies were also subsequently performed in other hosts [7,8], mechanistic insight into successful recombinant protein production has remained elusive.

Building on our previous transcriptome analysis [3], we show here how we identified high-yielding strains for the well-characterized [9-11] eukaryotic glycerol facilitator, Fps1, which is a non-trivial production target for further structural study. Specifically, we characterized *spt3Δ*, *srb5Δ *and *gcn5Δ *as effective production hosts for Fps1, where the yield improvement was up to a factor of 9 over the corresponding wild-type control. Improved yields of Fps1 were not explained by changes in promoter activity or *FPS1 *transcript number, but a post-transcriptional mechanism was suggested by the observation that each strain had elevated levels of *BMS1 *transcript compared to wild-type, as Bms1, the gene product of *BMS1*, is involved in ribosome biogenesis [12]. Subsequent overexpression of *BMS1 *in a doxycycline-dependent manner revealed that maximal membrane protein yield is correlated with an optimum level of *BMS1 *transcript for Fps1 and can be specifically tuned to maximize yields of other functional membrane (human adenosine 2A receptor) as well as soluble (green fluorescent protein) protein targets. By altering the amount of *BMS1 *transcript, the metabolism of high-yielding cultures changed substantially as determined by on-line flow microcalorimetry. This coincided with the ratio of 60S and 40S ribosomal subunits being perturbed, which we propose is the key to maximizing recombinant protein yields. This work demonstrates the power of a rational approach to recombinant protein production by finally offering an insight into the actual mechanisms involved.

## Results

### Three host strains deleted for genes involved in transcriptional regulation give substantially higher Fps1 yields than the wild-type parent strain

43 deletion strains were screened in shake-flasks to test the effect of genes that we had previously shown to be down-regulated under high-yielding production conditions of our target protein, Fps1 [3], or that were known to be from related pathways, especially in cases where a deletion strain was non-viable. The amount of membrane-bound Fps1 from late-log phase shake-flask cultures was quantified from immunoblots relative to the wild-type yield, with all immunoblot signals being below saturation. A cut-off point twice that of the wild-type was set for selecting strains for further study resulting in three strains being analyzed in bioreactors. The three deleted genes are known to be components of the transcriptional SAGA (*GCN5*, *SPT3*) and mediator (*SRB5*) complexes [13]. It has been hypothesized that SAGA may have a role in the transcription of 10% of genes, most of which seem to be stress-induced and that this might reflect the need to balance inducible stress responses with the steady output of housekeeping genes [14]. In contrast, the mediator complex appears to be required for all transcriptional events and transmits regulatory signals from transcription factors to RNA polymerase II, interacting directly with the unphosphorylated carboxy-terminal domain of RNA polymerase II, forming part of the pre-initiation complex, thus stimulating transcription [15].

**Figure 1:**
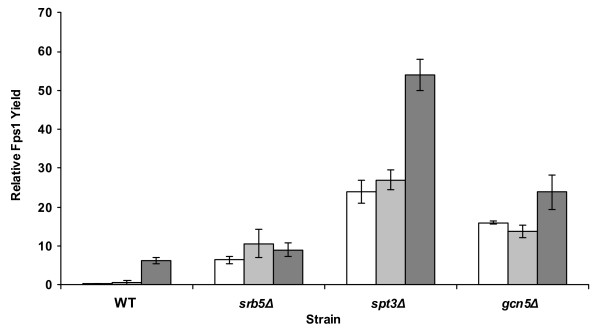
**Analysis of yields of Fps1 from three deletion strains cultured in 2 L bioreactors**. Fps1 yields are reported relative to a control sample, which previously represented our highest Fps1 yield obtained [3], as described in the Methods section. The y-axis shows the factor improvement over the control. Maximum yields are indicated from four immunoblots from duplicate bioreactor cultures. Error bars represent the standard deviation (n = 4). White bars are the values for cells grown in CSM medium, pale grey for CSM medium supplemented with 10 μg/mL myo-inositol and dark grey bars for 2 × CBS medium.

Figure 1 summarizes the Fps1 overproduction data from bioreactors in three *S. cerevisiae *strains and demonstrates that the *spt3*Δ strain offers a factor of 9–69 medium-dependent increase in Fps1 yield compared with wild type, the *srb5*Δ strain a factor of 1–18 increase and the *gcn5*Δ strain a factor of 4–46 increase. The *spt3*Δ strain gave the highest final yield of a factor of 54 over our internal control. We also noted that the move from shake flask, where these strains were initially screened, to bioreactor had resulted in significant yield improvements for each strain relative to the wild-type: the greatest increase in yield compared to the wild-type strain in shake-flasks was only a factor of 5 for the *spt3*Δ and *srb5*Δ strains (data not shown). The choice of medium also had a role to play with the relationship between increased yield and medium composition being strain independent: 2 × CBS was optimal in all cases (Figure 1), although CSM revealed greater improvements in the deletion strains compared to wild-type. There was no correlation between increased biomass and Fps1 yield since the highest yield of Fps1 was observed in early-to-mid glucose phase samples, whereas the highest biomass was always achieved in the ethanol phase. All mutant strains grew to lower cell densities than the wild type and exhibited slow-growth phenotypes. The reported auxotrophy of *srb5Δ *for myo-inositol [16], was not a significant contributor to its phenotype under our experimental conditions with it consistently out-performing the wild type in Fps1 overproduction (Figure 1).

As the host strains were all deleted for genes involved in transcription, real time quantitative PCR (Q-PCR) was used to measure the levels of plasmid-borne *FPS1 *(Table 1) to determine whether this was correlated with the protein yields observed in Figure 1. Endogenous *TPI1 *levels were used as an indicator of *TPI1 *promoter activity, as plasmid-borne *FPS1 *was cloned under the control of the *TPI1 *promoter and it was assumed that the activities of this promoter in both contexts would be similar. Within error we noted that RNA levels for *FPS1 *were comparable even though the deletion strains apparently had lower promoter activity (Table 1). We thus concluded that changes in the yield of Fps1 were likely to be explained by post-transcriptional events, in line with our earlier observations [3].

**Table 1:** Fps1 yield correlates with *BMS1 *transcript number in the host cell

	**Copies mRNA/cell**		
**Strain**	***TPI1***	***FPS1***	***BMS1***
**wild-type**	53.6 (5.0)	60.1 (18.2)	0.1 (0.0)
***gcn5Δ***	15.4 (3.4)**	61.5 (3.0)	0.7 (0.2)**
***spt3Δ***	12.5 (1.8)**	64.9 (26.0)	0.6 (0.1)**
***srb5Δ***	38.2 (12.5)*	87.4 (1.5)	0.7 (0.2)**

Analysis of mRNA from shake-flask cultures was performed using real time quantitative PCR. The data were normalized using the reference genes *ACT1 *and *PDA1 *and the signal was scaled to mRNA copies/cell according to a SAGE study [32], in which copies of mRNA/cell of the reference genes had previously been determined. Standard deviation in parentheses was calculated for the samples (n = 4). A single asterisk denotes significance at the P < 0.05 level (paired 2-tailed T-test), and a double asterisk at P < 0.01 level.

### Up-regulation of *BMS1 *is a marker of high-yielding conditions

Using Q-PCR, we examined changes in genes that we had previously shown to be up- or down-regulated under high-yielding production conditions [3] or that were known to be from related pathways. From this, we noted a clear relationship between Fps1 yield and a factor of 6–7 increase in *BMS1 *transcript number over wild-type in all three host strains (Table 1). In order to assess whether up-regulation of *BMS1 *over wild-type levels is a marker of high-yielding strains, we screened three further strains, two giving wild-type- (*spt8Δ*, *med6Δ*) and one giving improved (yTHC*SRB6*, where *SRB6 *is over-expressed in a doxycycline-repressible manner) Fps1 production yields. Table 2 shows that only yTHC*SRB6 *had an increase in *BMS1 *transcript number over wild-type, confirming that *BMS1 *up-regulation over wild-type levels is a marker of improved yields.

**Table 2:** *BMS1 *up-regulation can be used as a marker for selecting high-yielding strains

**Strain**	**Copies *BMS1 *mRNA/cell**	**Fps1 yield relative to wild-type**
**wild-type**	0.1 (0.1)	100 (27.0)
***spt8Δ***	0.2 (0.0)	104 (28.0)
***med6Δ***	0.2 (0.1)	55.6 (15.0)
**yTHC*SRB6***	0.8 (0.3)**	310 (12.0)**

Analysis of mRNA was performed as described in the legend to Table 1, and of Fps1 yield as described in the Methods section. Standard deviation in parentheses was calculated for the samples (n = 4). A double asterisk denotes significance at the P < 0.01 level (paired 2-tailed T-test).*SPT8 *encodes a subunit of the SAGA complex, required for inhibition at some promoters. *MED6 *and *SRB6 *encode subunits of the mediator complex.

**Figure 2:**
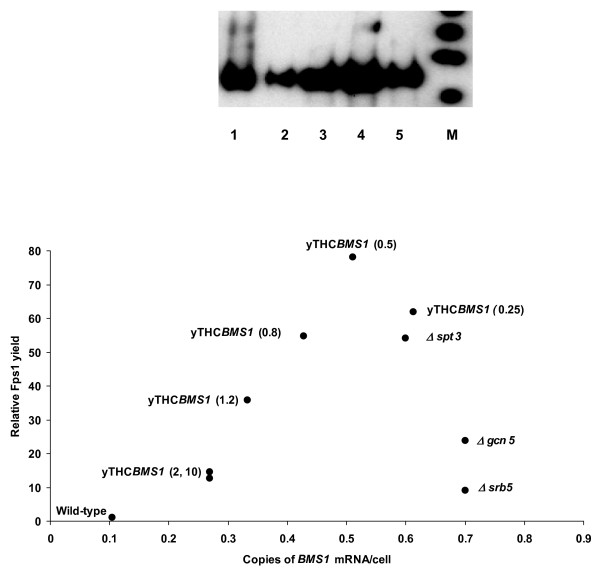
**Maximum yields of Fps1 are achieved by tuning *BMS1 *transcript number in a doxycycline-repressible system (yTHC)**. Fps1 yield in shake flasks is presented for the yTHC*BMS1 *overexpression strain cultured in 2 × CBS medium with varying amounts of doxycycline (the concentration in μg/mL is shown in parentheses next to each point), thereby varying the copies of *BMS1 *RNA/cell. The y-axis shows the factor improvement over the control, as for Figure 1. For comparison, data for the wild-type and the three deletion strains presented in Figure 1 (*srb5Δ*, *spt3Δ *and *gcn5Δ*) are also shown. Inset is a typical anti-HA-tag immunoblot showing Fps1 yields for 75 μg total wild-type membrane (lane 1), and 15 μg total yTHC*BMS1 *membranes extracted from shake-flask cultures grown in the presence of 0 (lane 2), 0.25 (lane 3), 0.5 (lane 4) and 10 μg/mL (lane 5) doxycycline. The markers (M) are 62, 98, 188 kDa ascending from the bottom of the gel upwards.

We next used a doxycycline-repressible *BMS1 *overexpression strain from the Open Biosystems yTHC collection (Thermo Fisher Scientific, UK) that allows gene expression to be tuned by the addition of doxycycline to the growth medium. This permitted further examination of the correlation between up-regulation of *BMS1 *and recombinant Fps1 yield. We found that in shake flask cultures, yield varied as doxycycline concentration (and hence *BMS1 *transcript number) was varied, as shown in Figure 2. Maximal Fps1 yields of a factor of 78 over wild-type were obtained at 0.5 μg/mL doxycycline. On culturing this strain in a bioreactor in the presence of 0.5 μg/mL doxycycline, the yield improvement over wild-type rose by up to a factor of 137 (data not shown). At 0.5 μg/mL doxycycline, the *BMS1 *level was 0.5 copies/cell (standard deviation = 0.1; n = 2) compared with that of 0.1 copies/cell in the wild type strain, then decreased with increasing doxycycline concentration (Figure 2).

### The *BMS1 *overexpression strain, yTHC*BMS1*, can be further tuned to improve the functional yield of the G-protein coupled receptor, hA2aR, and soluble green fluorescent protein

**Figure 3:**
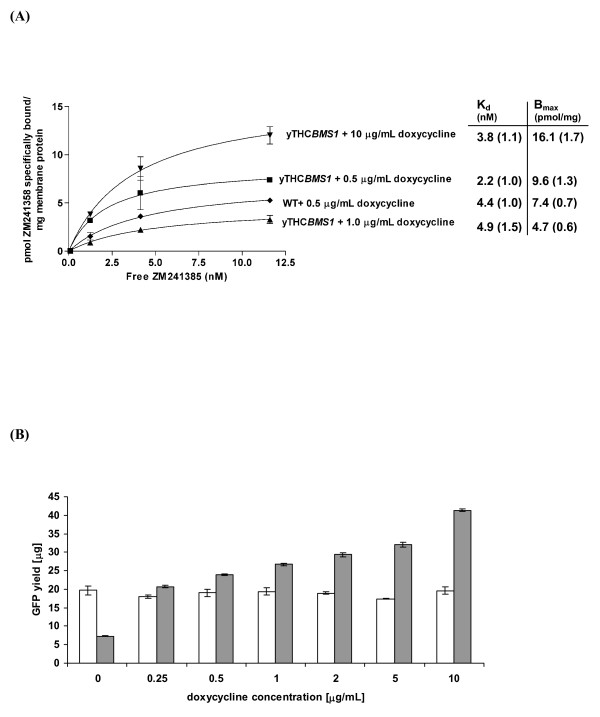
**The yTHC*BMS1 *strain, which produces the highest yields of Fps1, can also be used to improve the functional yield of other proteins**. (A) Binding of ZM241385 on yeast membranes producing the G-protein coupled receptor, hA2aR is shown for duplicate determinations. Error bars represent the standard deviation (n = 2). Also shown are the K_d _(nM) and B_max _(pmol receptor/mg protein) values with standard deviation given in parentheses (n = 2). (B) The yTHC*BMS1 *strain (grey bars) improves the functional yield of green fluorescent protein by a factor of 2 over wild-type (white bars). Error bars represent the standard deviation (n = 3). Shown is the total GFP yield from a 50 mL culture of OD_600 _1.0.

In order to examine whether our screening strategy using Fps1 as a target, had resulted in an Fps1-specific result, we tested the human G-protein coupled receptor, A2aR in our best-performing strain. Figure 3A shows that the improvement in active hA2aR as assessed by radioligand binding was small for cells cultured under conditions optimized for Fps1 production (0.5 μg/mL doxycycline). On examining whether hA2aR production could be tuned further, we confirmed that maximal binding activity was obtained at a doxycycline concentration of 10 μg/mL (where *BMS1 *levels were approximately three times those in wild-type cells). Analysis of the binding curves confirmed that this was due to an increase in receptor expression (i.e. the maximum amount of ZM241385 bound) and not because of any change in the affinity of the receptor for the radioligand. Furthermore, the strain could be tuned to maximize yields of functional green fluorescent protein, doubling wild-type yields at 10 μg/mL doxycycline when grown in 50 mL shake-flask cultures (Figure 3B). These results suggested that the up-regulation of *BMS1 *in a target-protein-specific manner might be the key to maximizing yields for a range of proteins.

### On-line flow microcalorimetry data indicate different metabolic activity in high-yielding versus low-yielding strains

**Figure 4:**
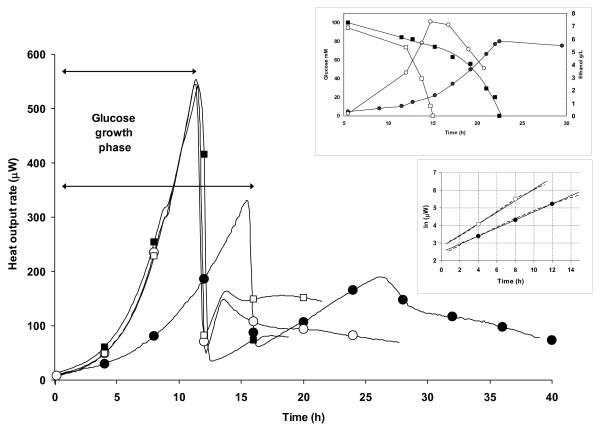
**Calorimetric profiles for the yTHC*BMS1 *and wild-type strains**. Profiles are shown for yTHC*BMS1 *producing Fps1 in the absence (white circles) or presence (black circles) of 0.5 μg/mL doxycycline. Those for the wild-type in the absence (white squares) or presence (black squares) of 0.5 μg/mL doxycycline overlay the former trace. The top inset panel shows the glucose (squares) and ethanol (circles) data for the yTHC*BMS1 *strain producing Fps1 in the absence (white symbols) or presence (black symbols) of 0.5 μg/mL doxycycline. The lower inset shows the natural log plot of the glucose phase data for the yTHC*BMS1 *strain, showing the linear fit used to calculate rate constants of 0.32 h^-1 ^and 0.21 h^-1 ^in the absence (white circles) or presence (black circles) of 0.5 μg/mL doxycycline, respectively.

We noted that high-yielding strains had lower growth rates than low yielding strains as previously observed in a wide range of cell-types, including engineered mammalian cells [17]. In order to assess whether this was accompanied by changes in metabolic activity, we used on-line flow microcalorimetry to measure the heat output of the recombinant protein-producing strains (Figure 4). The calorimetry traces in the glucose phase, where protein production peaks [3], were similar under all conditions except the maximal Fps1-yielding one (Figure 4). In the latter case, the trace mirrored the OD_600_-derived growth curve of yTHC*BMS1 *in the presence of 0.5 μg/mL doxycycline (Figure 4), consistent with the metabolic burden of increased recombinant protein production.

As expected, the heat outputs in all but the maximal Fps1-yielding conditions were indistinguishable. A natural log plot of the glucose growth phases for the low-yielding strains had highly similar pseudo-first order rate constants of 0.39 h^-1^, 0.37 h^-1^and 0.32 h^-1^, for the wild-type in the absence or presence of doxycycline, and yTHC*BMS1 *in its absence, respectively, yielding a mean growth rate of 0.36 h^-1 ^(where the standard deviation is 0.04). This is in excellent agreement with the mean growth rate of 0.34 h^-1 ^calculated from OD_600 _values and dry weights (Figure 4, lower insert) and is in line with typical growth rates for glucose-grown *S. cerevisiae *in bioreactors [18]. In contrast the mean growth rate for the maximal Fps1-yielding conditions (yTHC*BMS1 *in the presence of 0.5 μg/mL doxycycline) was calculated to be 0.21 h^-1 ^from triplicate determinations (standard deviation is 0.02), indistinguishable from 0.19 h^-1 ^calculated from OD_600 _values and dry weights (Figure 4). This is 58% of the mean growth rate for the low-yielding strains and is clearly reflected in the differing peak areas of the glucose growth phases (Figure 4): the corresponding heat yields for yTHC*BMS1 *in the presence and absence of 0.5 μg/mL were 5.4 kJ/g and 6.5 kJ/g, respectively. This suggested a substantially different metabolism in high-yielding compared to low-yielding cultures, consistent with the different ethanol profiles (Figure 4, upper insert).

### Maximum protein yield is accompanied by an altered 60S:40S subunit ratio in the host cell

**Figure 5:**
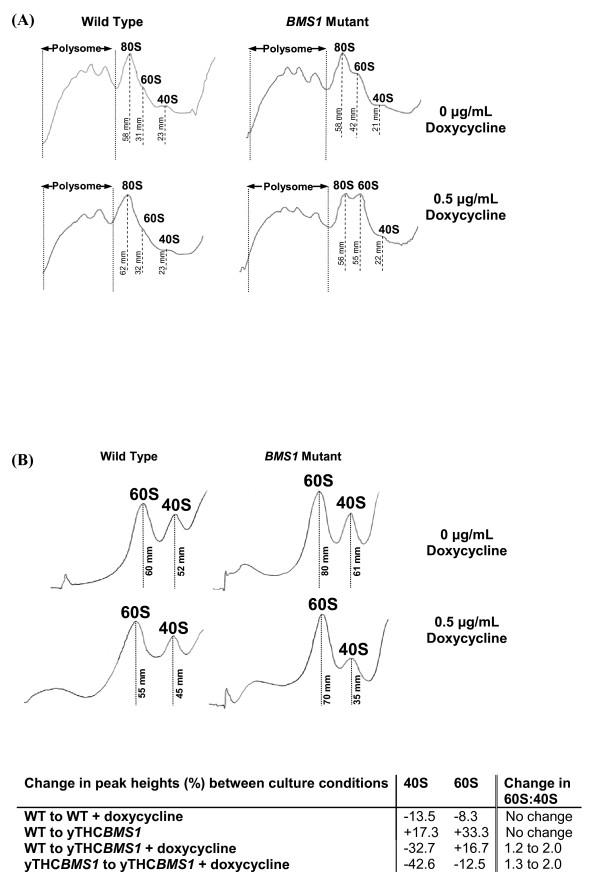
**Polysome profiles for the yTHC*BMS1 *and wild-type strains**. (A) Polysome OD_254 _profiles for yeast strains producing Fps1 in the absence (upper panels) or presence (lower panels) of 0.5 μg/mL doxycycline. Polysome peaks containing 2, 3 and 4 ribosomes are visible. (B) Ribosome disassociation profiles at OD_254 _for 50 mM EDTA-treated samples of wild-type and yTHC*BMS1 *strains producing Fps1 in the absence and presence of 0.5 μg/mL doxycycline (upper and lower panels respectively). Changes in peak heights and ratios of 60S and 40S subunits for all four conditions are tabulated.

We wished to identify a molecular event that might explain the apparently changed metabolism of high-yielding cells. As Bms1 has previously been reported to have a role in ribosome biogenesis [12], we therefore examined the polysome profile for yTHC*BMS1 *producing recombinant Fps1 in the presence and absence of 0.5 μg/mL doxycycline, the former condition giving maximal Fps1 yield compared to wild-type under the same conditions. As we had seen with the microcalorimetry traces, Figure 5A shows that the polysome profiles were similar under all conditions except the maximal Fps1-yielding one (lower right-hand panel) where elevated levels of free 60S subunits were clearly apparent. There was also an increased proportion of free 60S subunit for yTHC*BMS1 *in the absence of doxycycline (upper right-hand panel). These observations were supported when the ribosomes were disassociated with EDTA (Figure 5B): in the *BMS1 *overexpression strain, the relative proportion of 60S subunit was shown to be increased compared to wild-type in both the absence and presence of doxycycline. In relation to this, in the absence of doxycycline the Fps1 yield is increased by a factor of 10 over wild-type, while at 0.5 μg/mL it is increased by a factor of 78, suggesting that a change in the proportions of 60S and 40S subunits may result in an increased protein yield. Despite differences in the absolute peak heights, the ratio of 60S to 40S was similar in all traces in Figure 5B except the maximal Fps1-yielding one (lower right-hand panel). In this latter condition, the relative amount of 40S was lower than in all other conditions leading to a 2:1 ratio of 60S to 40S in comparison with a ratio of approximately 1:1 for all other conditions tested. Profiles for yTHC*BMS1 *cultured in 0, 0.25, 0.5, 1.0, 4.0 and 10.0 μg/mL doxycycline, showed that the relative proportion of 40S subunit decreased as expected [12] to below our detection limit with increasing doxycycline concentration, whilst the elevated level of 60S compared to wild-type remained essentially constant (data not shown). In agreement with this, 25S:18S ratios were 2:1 in the absence of doxycycline, in agreement with typical values for wild-type *S. cerevisiae *[19], but increased to 3:1, 5:1 and 7:1 at 0.5. 2.0 and 5.0 μg/mL doxycycline respectively.

## Discussion

A recent analysis of membrane protein production in *E. coli *[7] reported that increases in levels of chaperones and proteases were associated with increased membrane protein production and it was speculated that low yields were due to limited Sec translocon capacity. Our previous transcript analysis identified up-regulation of *SEC62 *in high yielding protein production. Sec62 is an essential subunit of the Sec63 complex (Sec63, Sec62, Sec66 and Sec72) and with the Sec61 complex, Kar2/BiP and Lhs1 forms a channel competent for SRP-dependent and post-translational SRP-independent protein targeting and import into the ER [20]. We found a *SEC63 *over-expression strain from the Open Biosystems yTHC *S. cerevisiae *collection did not give improved recombinant protein yields. Additionally, we found that *SRP102*, which encodes the signal recognition particle receptor β subunit, was down-regulated in high-yielding experiments and again that a *srp102Δ *strain gave only wild-type yields of Fps1. Whilst this does not preclude the eukaryotic secretory pathway from having a limiting effect in *S. cerevisiae *translational efficiency it does highlight clear differences between prokaryotic and eukaryotic cells.

We found instead that a common theme in our high-yielding strains was the up-regulation of *BMS1 *by a factor of 6–7 over wild-type (Table 1) suggesting the importance of ribosome biogenesis in maximizing production yield. Bms1 is an essential nucleolar protein that is evolutionarily conserved throughout the eukaryotic kingdom and has been suggested to have a regulatory role in the biogenesis of the 40S subunit [12] as well as being a GTP-binding protein [21]. Further experiments have led to the current model of Bms1 binding to the product of a second essential gene, Rcl1, in a GTP-dependent manner and shuttling Rcl1 to pre-ribosomes via its affinity for U3 snoRNA [22]. Our unpublished data suggest that over-expression of *RCL1 *in a doxycycline-dependent manner does not lead to improved yields, although this could be a result of Bms1 being limiting in a Bms1/Rcl1 complex. Nonetheless, recombinant protein translation was clearly enhanced by the overexpression of *BMS1 *alone (Figure 2).

Interestingly, it has previously been reported that mice in which deletion of both genes for ribosomal protein S6 led specifically to a lack of 40S synthesis, but normal 60S synthesis, in the liver survived for several weeks. Their livers could respond to fasting and re-feeding cycles, in which the mass of the liver nearly doubles. However, whereas partial hepatectomy in normal animals leads to rapid re-growth and cell division, the livers of mice defective in the production of 40S ribosomal subunits did not re-grow and showed no signs of cell division [23]. These results imply that a lack of 40S ribosome biogenesis can induce a checkpoint control that prevents cell cycle progression. This is entirely consistent with our observed slowing of growth that accompanied high-yielding protein production which has been widely reported by others as a feature of successful protein production hosts [24] and measured by on-line gas analysis [25]. Our own data support an interpretation that the yTHC*BMS1 *strain at 0.5 μg/mL doxycycline (high yielding conditions) has low fermentative activity, since it produces 16% less ethanol than the wild-type strain under the same conditions. Dry weights in the high yielding condition were also 6.5% lower than in poor yielding conditions over a full growth curve (122 h) in agreement with the observation that 1 g ethanol leads to 0.25 g dry weight in a typical yeast fermentation [26], and so 16% less ethanol should lead to 4% less dry weight. Furthermore the yTHC*BMS1 *strain had a lower average exponential phase RQ in the presence of 0.5 μg/mL doxycycline (RQ = 3.6) than in its absence (RQ = 4.1; in excellent agreement with literature values for wild-type strains grown on glucose [18]).

These observations are supported by our on-line flow microcalorimetry data. Kemp and co-workers were amongst the first to apply this technique to recombinant systems [17] and recently Gill and colleagues reported 'thermal profiling' as a method for on-line analysis of the growth of *E. coli *cultures expressing cyclohexanone monooxygenase under the control of an L-arabinose promoter in a multi-well format [27]. In the study presented here, we took the next step by using thermal profiles to rationalize the underlying mechanisms at work in our high-yielding cultures. The reduction in heat yield we observed suggests that high-yielding cells have a more efficient metabolism than low-yielding cells. As ribosome biogenesis is a major consumer of cellular energy resources [28] and its regulation is intimately linked to cell size, which in turn affects cell growth [29] as well as influencing other features such as the way that cells respond to stress [30], we hypothesized that the mechanism underlying this changed metabolism could be due to changes in ribosomal composition. The polysome profile for the high-yielding strain in which *BMS1 *is overexpressed in the presence of optimal levels of doxycycline for maximal Fps1 yield (at 0.5 μg/mL, Figure 5A) showed elevated levels of 60S subunits without any significant decrease in the levels of 80S or polysome. On further probing this by disassociating the ribosomes with EDTA, it could be seen that the ratio of 60S to 40S had been perturbed from wild-type levels of close to 1:1 to 2:1. This might suggest a previously unreported role (direct or indirect) for Bms1 in 60S biogenesis and also the possibility of an underlying mechanism in which binding and release of the 60S ribosomal subunit might be rate-limiting. We speculate that perturbing the ratio of the ribosomal subunits is key to maximizing recombinant protein yields by making protein production a more efficient cellular process. This might be influenced by an altered dynamic equilibrium between the transcript:40S complex and the 60S subunit which is available in greater amounts in the high-yielding strain. Of course it is also possible that some other component is affected by the perturbed 60S:40S ratio; both possibilities are the subject of our ongoing investigations.

## Conclusion

Our data show that recombinant protein production can be rationalized, guided by the results of transcriptome analysis of the host strain response. We specifically identify *BMS1 *as a gene whose expression can be tuned to facilitate high yielding protein production experiments. Under these conditions, there is a perturbation in the ratio of ribosomal subunits and the metabolism of the host cell is more efficient with respect to protein production.

## Methods

### Vectors

The *FPS1 *gene was tagged at its 3' end replacing the carboxy-terminal threonine residue with a sequence encoding three HA epitopes to permit immunodetection: SGRIFYPYDVPDYAGYPYDVPDYAGYPYDVPDYAAQCGR. The HA sequences are underlined. The construct was expressed from the *TPI *promoter in the 2 μpYX212 and pYX222 vectors (Novagen; now discontinued) which contain the *URA3 *and *HIS3 *selection markers respectively. The gene was cloned into the *Bam*H1 and *Hin*dIII sites and the vectors transformed into *S. cerevisiae *using the lithium acetate method. The GFP (GenBank U62636) and hA2aR [31] genes were amplified by PCR and cloned into the *Hin*dIII and *Xma*I sites and the *Hin*dIII and *Sal*I sites of pYX212 and pYX222, respectively. The GFP gene was cloned with an α-secretion factor to facilitate secretion into the culture medium.

### Yeast strains and culturing conditions

*S. cerevisiae *BY4741 is the parental strain for the deletion mutants *spt3Δ*, *srb5Δ *and *gcn5Δ *from the EUROSCARF collection  and the yTHC*BMS1 *strain (Open Biosystems) used in this study, and as such provided the wild-type control. Yeast cells were cultured in 2.5 L bioreactors containing 2 L of either CSM ± myo-inositol or 2 × CBS. CSM was composed of 1.7 g/L yeast nitrogen base (YNB) without amino acids, 5 g/L ammonium sulphate supplemented with 2% glucose ± 10 μg/mL additional myo-inositol, 2 × DO solution minus histidine or minus uracil (Clontech Yeast Protocols Handbook Version PR13103) and 10 mM MES pH 6. 2 × CBS was composed of 10 g/L ammonium sulphate, 6 g/L potassium dihydrogen phosphate, 1 g/L magnesium sulphate supplemented with 2% glucose, 2 mL/L each of trace element solution and vitamin stock solution (recipes shown below) and 2 × DO solution minus histidine or minus uracil. The pH was adjusted to, and maintained at, 6 via the online addition of 0.5 M NaOH. The agitation, aeration and temperature of the cultures were maintained at 700 rpm, 1 L per min and 30°C respectively. 1L trace element solution was composed of the following: 15 g EDTA, 4.5 g ZnSO_4_·7H_2_0, 1 g MnCl_2_·4H_2_O, 0.3 g CoCl_2_·6H_2_O, 0.3 g CuSO_4_·5H_2_O, 0.4 g Na_2_MoO_4_·2H_2_O, 4.5 g CaCl_2_·2H_2_O, 3 g FeSO_4_·7H_2_O, 1 g H_3_BO_3 _and 0.1 g KI. The pH was maintained at 6.0 with 1 M NaOH throughout the addition and finally adjusted to pH 4 with 1 M HCl prior to autoclave sterilisation and storage at 4°C. 1 L vitamin solution was composed of the following: 0.05 g D-biotin, 1 g Ca D (+) panthothenate, 1 g nicotinic acid, 25 g myo-inositol, 1 g thiamine hydrochloride, 1 g pyridoxol hydrochloride and 0.2 g D-amino benzoic acid. pH maintained at 6.5 with 1 M HCl. The vitamin solution was filter sterilized and stored as 20 mL aliquots at 4°C. Plasmid retention was verified by the plating of the cells onto CSM + inositol agar in the absence of histidine or uracil and incubation at 30°C for 4 days. To initiate each experiment, 50 mL of a given medium were inoculated with fresh yeast cells in a baffled shake flask and cultured for up to 72 h in a shaking incubator at 30°C, 220 rpm. This pre-culture was subsequently used to inoculate 200 mL of the same medium in a baffled shake flask and cultured under the conditions outlined above to an OD_600 _of 1. This was then used to inoculate the bioreactors to initial OD_600 _= 0.05. For the production of hA2aR, the strains BY4714 and yTHC*BMS1 *were transformed with hA2aR vectors and cultured in 1.75 L 2 × CBS supplemented with 10 mM theophylline and 0.5, 1.0 or 10.0 μg/mL doxycycline. The cells were harvested by centrifugation once the glucose concentration of the cultures was in the range 5 – 10 mM.

### Sampling, extracellular substrate determination and membrane preparation

Samples were withdrawn at various points in both the glucose and ethanol phases. 15 – 100 mL culture were centrifuged at 5000 × g, 4°C for 5 min. 0.5 mL of the supernatant was stored at -20°C for glucose and ethanol analyses. The cell pellets were frozen in liquid nitrogen and stored at -80°C for subsequent membrane preparation. Ethanol analysis (10176290035, R-Biopharm, Germany) was performed according to the manufacturer's instructions. Glucose concentrations were calculated with an Accu-Chek Active glucose analyzer (Roche Diagnostics, UK). Cell pellets were fractionated with glass beads (1:1 ratio) in 2 mL cell breaking buffer (50 mM potassium phosphate pH 7.4, 100 mM NaCl, 0.5 mM EDTA, 5% glycerol, 4 mM PMSF). The cells were agitated in a Fast Prep (Thermo Fisher Scientific, UK) at speed 6.5, employing 6 × 40 s pulses with 2 min incubations on ice between pulses. The samples were clarified at 10,000 × g, 4°C for 30 min and the total membrane pellet recovered from the supernatant at 100,000 × g, 4°C for 60 min. Total membranes were re-suspended in 50 μL of Buffer A (20 mM Hepes pH 8, 50 mM NaCl, 10% glycerol w/v) and the total protein concentration determined using a Bio-Rad (Hemel Hempstead, UK) Bradford-based assay with bovine serum albumin as standard. Dry-weight determinations were performed by collecting two samples of 5 mL by centrifugation for 5 min at 5,000 g. The cells were washed once in 5 mL water, dried for 24 h at 110°C, and stored in a desiccator for 24 h before being weighed. Typical dry weights were 2.1 g/L for low-yielding conditions and 2.0 g/L for high yielding conditions after completion of the glucose growth phase.

### Immunoblotting and yield analysis

30 – 75 μg of total membranes were loaded per lane on an 8% polyacrylamide gel and separated by SDS-PAGE at 150 V for 1.25 h. Proteins were subsequently transferred to a nitrocellulose membrane (ProTran; Geneflow, UK) at 100 V for 1 h. The membrane was blocked with phosphate-buffered saline (PBS) containing 5% milk overnight at 4°C before incubating with mouse monoclonal anti-HA (clone 12CA5; Roche Diagnostics, UK) at a 1:5,000 dilution in PBS/5% milk for 1 h at room temperature with gentle agitation. The membrane was subsequently washed twice with PBS/0.2% Tween 20 for 5 min before incubating with goat anti-mouse horseradish peroxidase-conjugated secondary monoclonal antibody (Sigma-Aldrich, UK) at a 1:5,000 dilution in PBS/5% milk for 1 h at room temperature with gentle agitation. The membrane was washed as above and developed using an enhanced chemiluminesence detection kit (Geneflow, UK) following the manufacturer's instructions and visualized with a Chemidoc (UVItech, UK). The signal from each lane was quantified using either UVIband or the ImageGauge programme and was expressed as the factor improvement over our internal control (which is the previously-reported reference yield of Fps1 per μg of total membrane [3]) and was corrected for the amount of total membranes loaded per lane.

### Radioligand binding assay

Harvested cells were re-suspended in 30 mL breaking buffer and disrupted at 30,000 psi for 10 min using an Avestin C3. The samples were clarified by centrifugation at 10,000 × g, 4°C for 30 min and total membranes recovered from the supernatant at 100,000 × g, 4°C for 60 min. Total membranes were re-suspended in 2.5 mL Buffer A, the protein concentration determined using a NanoDrop 1000 (Thermo Fisher Scientific, UK) and 0.5 mL aliquots stored at -80°C. Membrane bound hA2aR was then determined using a radioligand binding assay based on the protocol of Fraser [31]. Membranes at 0.5 mg/ml with 0.1 U of adenosine deaminase were incubated with varying concentrations of ^3^H ZM241385 for 60 min at 30°C and non-specific binding was defined by including 1 μM ZM241385 in the incubations. Assays were terminated by centrifugation at 14,000 rpm in a bench-top centrifuge for 5 min. The supernatant was discarded, the pellets washed superficially with water and solublilized with Soluene, which was added to scintillation fluid and then counted to determine bound radioactivity. Binding was analyzed using PRISM Graphpad v 4.0 to determine K_d _and binding capacity.

### GFP fluorescence measurements

50 mL of 2 × CBS medium were inoculated with individual yeast colonies transformed with the GFP vector and cultured for up to 48 h in the presence or absence of various doxycycline concentrations at 220 rpm and 30°C. These cultures were used to inoculate fresh 50 mL 2 × CBS medium to a final OD_600 _of 0.1 and were cultured as above for 16 – 20 h. 1 mL samples were withdrawn and the cells pelletted at 5,000 × g, 4°C for 5 min and the supernatant collected. 200 μL supernatant were loaded in triplicate in a black Nunc MaxiSorp 96-well plate and the fluorescence recorded on a SpectraMax Gemini XS plate reader (Molecular Devices, Wokingham, UK) with excitation and emission wavelengths of 390 nm and 510 nm respectively, and a cut-off of 495 nm. Doxycycline fluorescence accounted for less than 5% of the signal up to concentrations of 10 μg/mL.

### RNA preparation and real time quantitative PCR

Yeast cells (60 mL) from two biological replicates and two technical replicates were harvested and frozen in liquid nitrogen. Total RNA was then prepared using the RNeasy kit from Qiagen with on-column DNAse treatment, following the manufacturer's instructions. Analysis of mRNA was performed using real time quantitative PCR (Q-PCR). 1.1 μg RNA was used in the cDNA reaction using the iScript cDNA Synthesis Kit (Bio-Rad, UK). Each sample was amplified using up to 30 cycles (20 s 94°C; 20 s 60°C; 20 s 72°C) in a Bio-Rad iCycler iQ, and the data were analyzed using iCycler IQ version 3.0. The data were normalized using the reference genes *PDA1 *and *ACT1 *and the signal was scaled to mRNA copies/cell according to a SAGE study [32] in which copies of mRNA/cell of all the reference genes had previously been determined.

### On-line flow microcalorimetry

Flow microcalorimetric data were collected for bioreactor-grown cultures as detailed above, in the absence or presence of 0.5 μg/mL doxycycline using a Thermal Activity Monitor 2277 (Thermometric AB, Sweden) with a flow vessel working volume of 0.6 cm^3^. The system was sterilized by successively pumping through solutions of sterile deionized water, 70% ethanol (v/v), sodium hydroxide (0.5 M) and finally sterile deionized water. The thermostatic water bath was maintained at 30°C, a sampling interval of 600 s was chosen in the Digitam v4.1 software and the pumping rate was set at 48 mL/h (Watson Marlow 400–403 u/VM2, 30 rpm). The calorimeter was calibrated at the same temperature, flow rate and amplifier setting (1000 μW) to be used during experimental data collection in order to establish a steady baseline deflection. Electrical calibration was done in the culture medium. Once the baseline was established, the inlet and outlet tubes were placed in the bioreactor in the presence of 70% ethanol prior to inoculation in order to maintain sterility. Power-time data were imported into Microcal Origin 7.0 where all subsequent data analysis was performed. Pseudo 1^st ^order rate constants were extracted from the calorimetric data using the equation below [33], for a flowing system that conforms to 1^st ^order kinetics.

$$\Phi =-FCH(1-e^{-k_{1}\tau })e^{-k_{1}t}$$

where Φ is the calorimetric output (Js^-1^); F is the flow rate (dm^3^s^-1^); C is the concentration of reactant (mol dm^-3^); H is the enthalpy of reaction (kJ mol^-1^); k is the rate constant (s^-1^); τ is the residence time of reacting solution (s); t is time (s). By plotting the natural logarithm of power against time, a pseudo 1^st ^order rate constant for exponential growth under the conditions employed was obtained. This was validated against the value calculated from OD_600 _and dry weight measurements. Peak areas were calculated using MicroCal Origin 7.0 using the calculus function embedded in the software. Integration limits were chosen to omit the initial 10 min of data to minimize errors associated with the initiation of the experiment. The high limit was taken as being the very bottom of the down-curve of the glucose peak.

### Polysome profiling

50 mL of 2 × CBS medium were inoculated with individual yeast colonies and cultured for up to 72 h in the presence or absence of 0.5 μg/mL doxycycline at 220 rpm and 30°C. These cultures were used to inoculate fresh 50 mL 2 × CBS medium to a final OD_600 _of 0.1 and were cultured as above for 24 h. 50 mL 2 × CBS were inoculated to a final OD_600 _of 0.1 from these cultures and grown as stated above to a final OD_600 _of 1. Cycloheximide was then added to the cultures to a final concentration of 10 μg/mL and incubated for 10 min. Cells were recovered at 5,000 × g, 4°C, 5 min, stored on ice and then suspended in 0.4 mL lysis buffer (10 mM Tris-HCl pH 7.4, 100 mM NaCl, 30 mM MgCl_2_, 200 μg/mL heparin, 50 μg/mL cycloheximide, 1 mM DTT, 1 μl/mL RNase inhibitor and one EDTA-free protease inhibitor cocktail per 10 mL buffer) prepared in fresh DEPC-treated water with an equivalent volume of acid-washed glass beads. The cells were then disrupted in a Precellys 24 (Bertin Technology, France) at a speed of 6,500 rpm for 10 s. Samples were cooled on ice for 2 min followed by 2 additional rounds of disruption. The samples were clarified twice at 5,000 × g, 4°C, 5 min and 10,000 × g, 4°C, 20 min and the supernatant recovered. The RNA was subsequently quantified at 260 nm on a Nanodrop (Thermo Fisher Scientific, UK) and 5 – 10 OD_600 _units loaded on a freshly-prepared 10 mL 10 – 50% sucrose gradient. Gradients were ultracentrifuged at 37,000 rpm, 4°C in a SW40 Beckman rotor for 2 h 40 min. The OD_254 _profile of the gradients were recorded by chart recorder (Pharmacia LKB REC 102, GE Healthcare Life Sciences, UK) by passing the gradient (from the bottom of the tube to the top of the tube) through an AmershamPharmacia UV detector (GE Healthcare Life Sciences, UK) at a speed of 1.8 mL/min with the simultaneous collection of 0.7 mL fractions. These fractions were stored at -20°C for later analysis. For EDTA polysome disruption profiling, yeast cultures were prepared as above and the cells recovered in the absence of cycloheximide. Cells were resuspended in 0.4 mL lysis buffer lacking cyclohexamide, and disrupted and clarified as above. The RNA was quantified as previously stated and 10 OD_600 _units were mixed with 50 mM EDTA and loaded on top of freshly-prepared 10 mL 7.5 – 20% sucrose gradients. The gradients were then ultracentrifuged and processed as described above.

### Analysis of ribosomal RNA

50 mL 2 × CBS medium were inoculated with individual yeast colonies transformed with the Fps1-HA_3 _vector and cultured for up to 48 h in the presence or absence of various doxycycline concentrations at 220 rpm and 30°C. These cultures were used to inoculate fresh 50 mL 2 × CBS medium containing various doxycycline concentrations to a final OD_600 _of 0.1 and were cultured as above to a final OD_600 _of 1 – 3. Cells from 0.5 mL culture were collected by centrifugation at 1,000 × g, 4°C for 5 min and the supernatant discarded. The pellet was then processed and the RNA collected with an RNeasy Mini Kit (Qiagen) according to the standard enzymatic lysis protocol for yeast. The RNA was quantified and samples diluted to 150 ng/μL. The RNA samples were heated to 70°C for 3 min prior to loading 150 ng samples in triplicate on an Agilent RNA 6000 Nanochip and running on an Agilent Bioanalyzer 2100 according to the manufacturer's instructions.

## Competing interests

The authors declare that they have no competing interests.

## Authors' contributions

NiB and RD were involved in all aspects of the experimental design, data collection, analysis and interpretation. LG participated in collecting the bioreactor data. NaB optimized and performed the microcalorimetry experiments. JW and SB performed the polysome profiling experiments. DP performed the binding assays. MO analyzed the microcalorimetry data. RB initiated the study, coordinated the data collection, analysis and interpretation, and drafted the manuscript. All authors contributed to the final version of the manuscript.
